# Synergistic Effects of Zn-Rich Layered Double Hydroxides on the Corrosion Resistance of PVDF-Based Coatings in Marine Environments

**DOI:** 10.3390/polym17030331

**Published:** 2025-01-25

**Authors:** Hissah A. Alqahtani, Jwaher M. AlGhamdi, Nuhu Dalhat Mu’azu

**Affiliations:** 1 Department of Chemistry, College of Science, Imam Abdulrahman Bin Faisal University, Dammam 31451, Saudi Arabia; 2 Department of Environmental Engineering, College of Engineering, Imam Abdulrahman Bin Faisal University, Dammam 31451, Saudi Arabia; nmdalhat@iau.edu.sa

**Keywords:** polymer, layer double hydroxide, nanocomposite coatings, Zn LDH, corrosion

## Abstract

In this study, zinc–aluminum layered double hydroxide (ZLDH) and its calcined counterpart (CZLDH) were synthesized and incorporated into a poly(vinylidene fluoride) (PVDF) matrix to develop high-performance anti-corrosion coatings for mild steel substrates. The structural integrity, morphology, and dispersion of the LDH fillers were analyzed using FTIR, XRD, Raman spectroscopy, and SEM/EDS, while coating performance was evaluated through water contact angle (WCA), adhesion tests, and electrochemical techniques. Comparative electrochemical impedance spectroscopy (EIS) and potentiodynamic polarization tests in a 3.5% NaCl solution revealed that the ZLDH/PVDF coating exhibited superior corrosion resistance and long-term stability compared to CZLDH/PVDF and pristine PVDF coatings. The intact lamellar structure of ZLDH promoted excellent dispersion within the polymer matrix, enhancing interfacial adhesion, reducing porosity, and effectively blocking chloride ion penetration. Conversely, calcination disrupted the lamellar structure of ZLDH, reducing its compatibility and adhesion performance within the PVDF matrix. This study demonstrates the critical role of ZLDH’s structural integrity in achieving enhanced adhesion, barrier properties, and corrosion protection, offering an effective anti-corrosion coating for marine applications.

## 1. Introduction

Research on metal corrosion remains a priority in the scientific community because of security concerns and significant financial implications. Organic coatings are the most commonly used solution to protect metallic materials from corrosion in industrial and marine environments [1]. Polymer coatings act as barriers to prevent direct contact between metal substrates and aggressive environments. Nevertheless, polymer coatings undergoing the curing process inevitably experience shrinkages and internal defects due to the solvent’s volatilization [2]. Traditional coatings fail to offer adequate long-term protection for metal surfaces due to their structural flaws, which allow corrosion agents to easily penetrate the coating and result in both coating deterioration and metal corrosion. Hence, there is a pressing requirement to enhance the anti-corrosive abilities of conventional polymer coatings [3]. These polymer-based coatings form a strong barrier that effectively blocks corrosive elements and can be easily enhanced with various additives, including anti-corrosive pigments, to further improve their protective properties [4]. To ensure a specified service life, organic coatings typically need to deliver (i) strong adhesion to the substrate, (ii) effective barrier properties against ions and water, and (iii) corrosion inhibition [5].

Chromate has long been recognized as one of the most effective chemical conversion coatings for providing high-efficiency corrosion protection. However, due to its environmental hazards, chromate has been increasingly banned across various industries [6]. Therefore, it is essential to develop new eco-friendly coatings that offer enhanced corrosion protection and long-term stability [7]. In response, alternative eco-friendly chemical conversion coatings, such as titanium [8], molybdate [9], zinc [10], and phosphate [11], have been introduced. Unlike chromium-based coatings, these environmentally friendly coatings often exhibit microscale cracks and lack self-healing properties, significantly limiting their effectiveness in protecting mild steel alloys [12]. However, in order to enhance its protective capabilities, the organic coating can receive cathodic protection by incorporating active metal particles. Scientists found in the early 1930s that incorporating zinc powder into an organic coating could offer sufficient cathodic protection even if the surface coating was slightly damaged [13,14]. In addition, sacrificial coatings are usually coatings rich in zinc. When corrosion happens, zinc particles serve as a sacrificial anode to shield the propeller (cathode) by turning into zinc oxide, which then covers the corroded area, changing the protection method from sacrificial to barrier protection [15]. The improved protective performance of zinc-rich coatings (ZRC) is primarily due to the enhanced electrical connection between zinc particles and substrate, the creation of a labyrinth effect by conductive fillers, and the reduction in coating porosity, among other factors. Nonetheless, there are varied findings regarding the impact of conductive fillers on the protective properties of ZRC [16].

Layered double hydroxides (LDHs) are a type of two-dimensional clay material composed of metal hydroxides arranged in a layered structure. Pigment production uses a variety of metal cations, including ZnAl and MgAl [17,18]. This distinctive configuration allows for the tuning of metal cations within the layers and the exchange of anions between them, enabling adjustments to the electrical conductivity of the resulting composites [19]. Upon calcination, the LDH structure undergoes a transformation where the intercalated anions and water molecules are removed, resulting in a highly porous material that enhances the surface area and exposes metal oxide sites. This change in structure alters the physical and chemical properties, including the increased thermal stability and enhanced surface reactivity of LDHs. This process alters the original layered double hydroxide structure, converting it into a combination of metal oxides and spinel phases, as confirmed by XRD analysis [20]. The functional groups in non-calcined LDHs, such as hydroxyl groups, play a crucial role in their interaction with coatings, whereas the calcined form provides a more reactive surface for interaction with corrosion inhibitors and enhances the overall performance of the coatings in terms of corrosion protection [21]. As chloride is favored by organic molecules in anion exchange, pigments containing hydrotalcite or calcined hydrotalcite may be used as chloride scavengers. Of all the nanomaterials, LDH nanocontainers have the capacity to store and release corrosion inhibitors while also showcasing a characteristic two-dimensional lamellar structure. This feature enables LDHs-based polymer coatings to possess both active and passive protective properties. The influence of zinc in the LDH array showed a noticeable effect on corrosion prevention when compared to magnesium with equal LDH ratio and quantity in the polymeric coating system [22]. However, because of the inherent structural features of LDH nanoflakes, they have a tendency to strongly clump together when added to the coating resin. The uneven distribution of LDH nanoflakes in coatings is primarily due to their dense clustering, posing a challenge in their use for polymer coatings [3].

Incorporating LDH structure with PVDF is a promising way to achieve more uniform dispersion in the coating matrix. In fact, polar dispersant solvents are capable of creating hydrogen bonds with the LDH galleries, enabling a significant amount of solvent molecules to penetrate the interlayer space and speeding up the delamination procedure [23]. Becker et al. have reported a method to disperse MgAl-LDHs into nanosheets throughout the polymer matrix using different organic solvents. Out of all the solvents analyzed, dimethylformamide DMF has the most significant amount of highly electronegative components: one oxygen atom and one nitrogen atom per molecule. This suggests that DMF has the greatest potential to form hydrogen bonds and consequently has the highest dielectric constant. DMF was utilized as a dispersing agent, leading to improved dispersion facilitated by this solvent, as confirmed by XRD analysis [24].

This work aimed to develop anti-corrosion coatings based on the ZnAl–LDH composites suitable for preventing corrosion on organic-coated steel surfaces. A pigmentary form of synthesized particulate material was synthesized and analyzed for incorporation into paint compositions. Additionally, the use of non-toxic zinc could further enhance the appeal of low-cost raw materials. XRD, FTIR, and SEM were used to analyze the morphology, composition, and structure of the fabricated LDH campsites and the coatings. The study focused on investigating corrosion protection by electrochemical impedance spectroscopy (EIS) and potentiodynamic polarization curves (PDP). The potential of ZnAl-LDH intercalated with an inorganic corrosion inhibitor was examined based on the collected data. Unlike previous studies, this work provides new composite coatings using ZnAl-LDH and compares the performance of the non-calcined form with its calcined counterpart. By comparing the two forms, we aim to explore the impact of these structural and functional differences on the performance of the resulting composite coatings. This comparison offers valuable insights into their efficacy as anti-corrosion coatings, an area not extensively explored in prior research. This research has the potential to aid in the advancement of novel systems for preserving metallic structures from corrosion, which excel in the cost-effective and secure management of waste during production and disposal.

## 2. Materials and Methods

### 2.1. Materials

Low carbon steel samples (AISI 1004) with dimensions (18.5 mm × 18.5 mm × 2.5 mm) were purchased from Co. Munford (Munford, TN, USA). Al(NO_3_)_3_·9H_2_O, Zn(NO_3_)_2_·H_2_O (analytical pure), and sodium chloride were purchased from LOBA Chemie (Mumbai, India). N,N-Dimethylformamide (DMF, ACS reagent, ≥99.8%) and Poly(vinylidene fluoride) (PVDF, average Mw ~ 534,000 by GPC, powder) were procured from Sigma-Aldrich (St. Louis, MO, USA). Sodium hydroxide and sodium nitrate were obtained from PanReac AppliChem (Barcelona, Spain). All the analytical grade chemicals were used as received without further purification. The deionized water was used in all the experimental processes.

### 2.2. Synthesis of the ZLDH and CZLDH Powder

**Figure 1:**
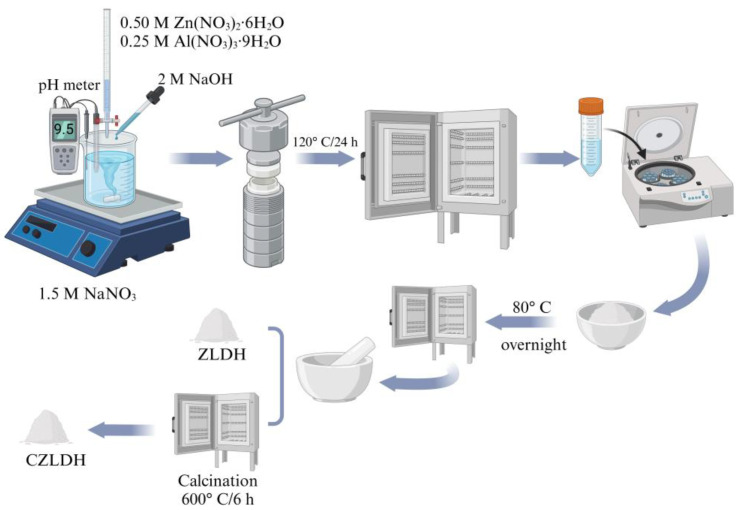
Illustration of the experimental procedure of synthesizing LDH composites.

In this paper, ZnAl-LDH (ZLDH) was synthesized using a combined coprecipitation route and hydrothermal treatment similar to earlier studies [22,25]. Here, 1.5 M sodium nitrate was liquefied in 100 mL of deionized water (DIW) at a constant pH = 9.5, adjusted simultaneously by 2 M NaOH, and while the obtained solution was stirred vigorously at room temperature, the other solution containing 0.5 M zinc salt and 0.25 M aluminum salt (Mg^2+^/Al^3+^ molar ratio equal to 2) in 50 mL of DIW was slowly added to this solution. Then, with sodium hydroxide (2 M), the pH of the attained solution was adjusted at 9.5 ± 0.5 by drop-wise addition for 45 min. It should be mentioned that all steps of the process were performed under a nitrogen-bubbling atmosphere. Then, the white suspension was sealed into a 200 mL Teflon-lined autoclave and heated at 120 °C for 24 h. The product naturally cools down to room temperature in the autoclave to obtain ZLDH hydrogel. Finally, the attained precipitate was centrifuged, washed thoroughly with deionized water several times to remove excess salts, and dried in an oven at 80 °C overnight. A part of the obtained ZLDH was calcined at 600 °C for 6 h to obtain CZLDH by transferring the layered double hydroxide (LDH) into layered double oxide (LDO). Figure 1 clearly presents the production process.

### 2.3. Preparation of the P/PVDF, ZLDH/PVDF, and CZLDH/PVDF Coatings

**Figure 2:**
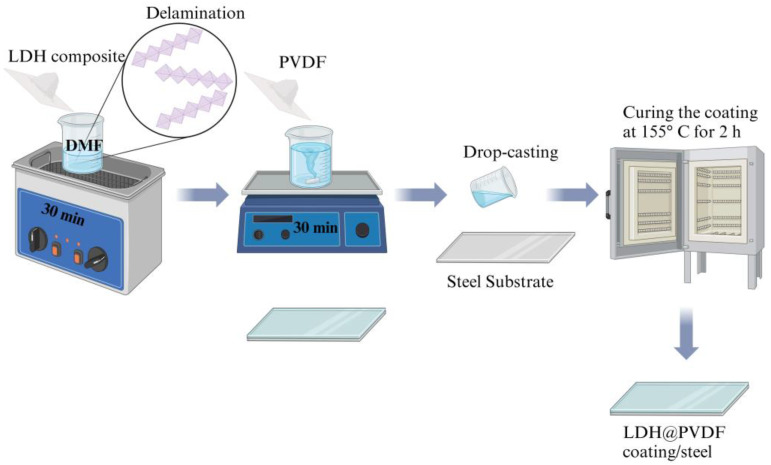
Schematic illustration of the preparation of LDHs/PVDF coatings.

Briefly, 2 wt% of ZLDH or CZLDH powder was first dispersed and exfoliated in 10 g of DMF through sonication waves for 30 min. Then, a certain amount of PVDF (0.8 g) was added to the exfoliated LDH-DMF homogenous suspension and stirred for 30 min at 40 °C. Before applying the composite coatings, the substrate surface was mechanically ground with SiC papers up to 1200 grit to ensure the surface roughness. Then, the substrate was cleaned with deionized water. Subsequently, the formed mixture was cast to the polished substrates to obtain uniform films and then cured in an oven at 155 °C for 2 h, as shown in Figure 2. For comparison, the pristine PVDF (P-PVDF) coating was prepared as in previous steps, excluding the addition of the LDH composites.

### 2.4. Characterization

The prepared samples were characterized through an FT-IR instrument (Shimadzu spectrophotometer, Model: IRAFFINITY-2, Kyoto, Japan) with KBr disks in the wavenumber range from 4000 to 400 cm^−1^ at room temperature with a resolution of 1 cm^−1^. The X-ray diffraction (XRD) patterns were recorded to study the crystal structure of the synthesized ZLDH and CZLDH by using (Shimadzu, Model: XRD-7000, Cu Kα radiating, voltage 40 kV and 30 mA, Kyoto, Japan) in the range of 2theta from 2° to 90° at ambient temperature and under atmospheric pressure. Raman spectra were used to analyze the element content and functional group of the synthesis ZLDH and CZLDH samples. The Raman spectra were recorded using a DXR-Raman Microscope (TESCAN, Model: VEGA3, Brno, Czech Republic). Scanning electron microscopy (SEM; TESCAN, Model: VEGA3, Brno, Czech Republic) equipped with an EDS detector was employed to investigate the surface and cross-sectional morphologies as well as the chemical compositions of the specimens. Static water contact angles were measured at ambient temperature by the falling drop method, and the volume of the water drop was about 10 µL. The water contact angles were measured by capturing images of the droplets on the coating surface using a high-resolution camera. The captured images were analyzed using dedicated software to determine the contact angles accurately.

In order to evaluate the adhesion of the coating, we utilized a cross-cut tape test, which consisted of using a cross-cut tester fitted with seven blades positioned at 1 mm intervals. Two orthogonal incisions were made to form a 36-block grid on the coated sample. Afterward, tape was put on the cross-cut spot and then peeled off at as close to a 180° angle as feasible, with adhesion tested following the ASTM D 3359 test method [26].

### 2.5. Electrochemical Measurements

All electrochemical experiments were carried out using a Gamry potentiostat system (Interface, Model 1000E, Gamry Instruments, Warminster, PA, USA) in a three-electrode cell, including a saturated calomel electrode (SCE) reference electrode, a platinum counter electrode, and the coated low carbon steel substrate as the working electrode with an exposed area of 1 cm^2^ at room temperature. Before measurements, OCP measurements were performed first, and EIS and Tafel measurements started only when the OCP values remained stable after 1 h, which allowed the system to stabilize. In addition, the EIS measurements were performed in 3.5 wt.% NaCl solution in different time periods (1 h, 1, 9, 18, and 30 days) with a sine signal with an amplitude of 10 mV v.s. OCP. The range of measured frequencies extended from 10^5^ Hz to 10^−1^ Hz, with a logarithmic sweep of 10 points per decade. The impedance plots were fitted using different equivalent circuits with the Gamry analysis software (https://www.gamry.com/support-2/software/). In order to ensure reproducibility, three parallel samples were used in each system. The potentiodynamic polarization curves were recorded with a sweep rate of 1 mV/s and within a scan range from −0.500 V to +1 V with respect to OCP.

### 2.6. Optimization of the ZLDH/PVDF Coatings for Corrosion Protection of Carbon Steel

The curing temperature, the curing time, and the amount of ZLDH were selected as important parameters to determine the best anti-corrosion coating. These parameters were performed sequentially. The curing temperature, the curing time, and the amount of ZLDH were changed, as shown in Table 1. Potentiodynamic polarization was operated with a sweep rate of 1 mV/s and within a scan range from +0.500 V to −0.500 V with respect to OCP to evaluate the corrosion protection properties of the coatings by experimenting with multiple conditions.

## 3. Results and Discussion

### 3.1. Structure and Morphology of ZLDH and CZLDH

#### 3.1.1. FT-IR Spectroscopy

**Figure 3:**
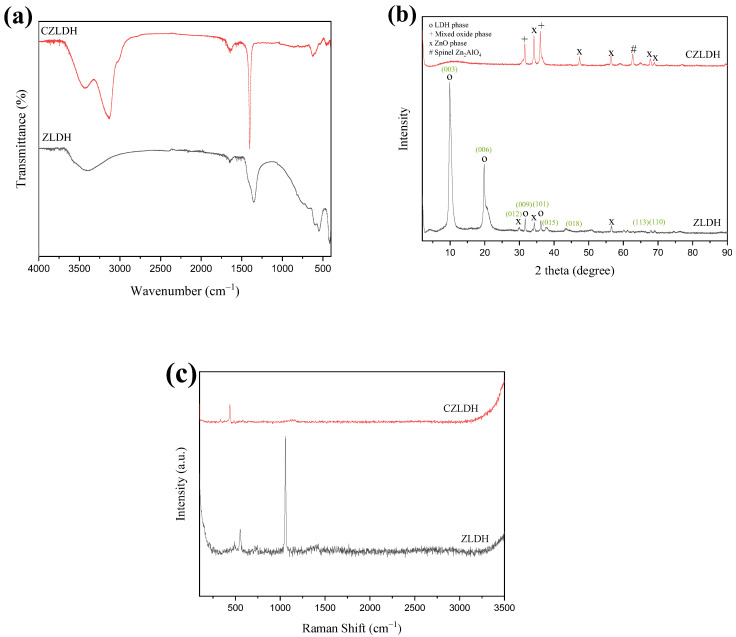
(**a**) FT-IR spectra, (**b**) XRD patterns, and (**c**) Raman spectra of the ZLDH and CZLDH powders.

Figure 3a shows the FTIR spectra of ZLDH and CZLDH powder composites. In the spectrum of ZLDH, a broad medium absorption band at about 3393 cm^−1^ is attributed to the stretching vibration of hydrogen-bonded hydroxyl groups (–OH) from both the brucite-like layers in ZLDH nanocomposite and the water molecules in interlayer [27]. The two adsorption peaks located at 1636 cm^−1^ and 1351 cm^−1^ are ascribed to the O–H bending vibration of the water molecules as a weak shoulder peak and stretching vibration of the nitrate ions in the interlayer spacing, respectively [28]. Generally, the peaks appearing from 400 to 700 cm^−1^ are because of (M–O, O–M–O, and M–O–M) bonds [29]. Therefore, the peaks at 595 and 547 are assigned to M–O and M-OH (M = Al, Zn) lattice vibrations in LDH layers [30]. After calcination of ZLDH, the adsorption band at 3500 cm^−1^ to 3000 cm^−1^ is ascribed to the stretching vibrations of surface-free hydroxyl groups [31]. The band at 1633 cm^−1^ belongs to water molecules present in the interlayer space. Moreover, the strong band at around 1400 cm^−1^ is assigned to the stretching vibration of residual bulk polydentate nitrate ions in interlayer spacing, which is attributed to the incomplete decomposition of the NO_3_^–^ groups in the LDH interlayer [32]. Though the ZLDH nanocomposite was destroyed after calcination, the yielded products still contain the interlayer nitrate anions and bound water. In addition, the band observed around 439 cm^−1^ corresponds to the O–M–O vibrational modes within the brucite-like layers, while the band at 609 cm^−1^ is associated with the lattice vibrational modes related to the translational movement of M–OH. The FT-IR results for CZLDH are also consistent with literature [33]

#### 3.1.2. XRD

The XRD patterns of the synthesized ZLDH and CZLDH nanocomposites are shown in Figure 3b. In the range of 2θ values between 4° and 35°, the most intensive peaks of the LDH diffractograms originate from diffraction on hydroxide layers [34]. In addition, the sharp and highly intense peaks indicate that the synthesized materials are fully crystalline and have an ordered structure [35,36]. The strong diffraction peaks at 2θ values 9.90°, 19.86°, 31.70°, 34.34°, 36.22°, and 56.52° can be ascribed to the (003), (006), (012), (015), (018), and (110) crystal planes of lamellar structure of ZnAl–LDH intercalated with nitrates [37], respectively. Moreover, the presence of a crystalline ZnO phase could explain the appearance of additional peaks [38]. This confirmed that the formed layer was indeed a part of the LDH phase, as in the literature [25,39]. After calcination of ZLDH at 600 °C for 6 h, it can be clearly seen that the diffraction peaks of (003) and (006) disappeared, revealing that the lamellar structure of the ZLDH was completely collapsed and converted into layered double oxide (LDO) and subsequently, new diffraction peaks were observed corresponding to phases of ZnO and ZnAl_2_O_4_ spinel [32]. On calcination at a moderate temperature (600 °C), the synthesized LDH nanocomposites are converted to mixed oxides. However, similar observations have been reported earlier for various calcined LDH samples [38,40,41]. Basal spacing for ZLDH nanocomposite is 8.7 A°, indicated by the (003) reflection, due to the water molecules still left after the low-temperature drying process, which is absent in CZLDH nanocomposite and consistent with what was reported previously [25,28,42]. However, the three diffraction peaks at 31.54°, 34.20°, and 36.02°, which corresponded to (100), (002), and (101), were more intense compared to the pattern of ZLDH, which indicates that the LDO zinc aluminate spinel (ZnAl_2_O_4_) crystalline phase is increased [37,43]. Further, new diffraction peaks were discovered at 47.34°, 56.46°, 62.68°, 67.78°, and 68.90°, which correspond to (102), (110), (103), (200), and (112) planes of CZLDH, respectively [32,44,45]. The characteristic peaks reveal the decomposition of the lamellar structure and the appearance of the spinel phase (ZnAl_2_O_4_), mixed oxide phase, and the ZnO phase. A well-formed crystalline zinc aluminate spinel phase was produced at a high annealing temperature of 600 °C. The mean crystallite size of nanocomposites was calculated by Sherrer’s equation based on XRD patterns (Equation (1)) [46].

D = Kλ/βcosθ(1)

where K, λ, β, and θ are the shape factors (0.90), the wavelength of X-rays (Cu, λ = 0.15406 nm), the FWHM in radians, and the position of the peaks in radians, respectively. The results exhibit that the crystallite size of ZLDH (15 nm) is smaller than the crystallite size of CZLDH (23 nm) (Table S1).

#### 3.1.3. RAMAN

Raman spectroscopy was carried out to obtain additional information regarding the elucidation of the crystal structure and the nature of gallery anions of the synthesized LDHs (Figure 3c) [47]. For the ZLDH sample, the appearance of a weak band at approximately 555 cm^−1^ can be attributed to the characteristic stretching vibration of the O–M–O band (M = Al, Zn) [48,49]. Additionally, the intensive peak centered around 1063 cm^−1^ is attributed to the interlayer nitrate anions in the pristine LDHs [47,49]. However, the M–O band was shifted to 429 cm^−1^ after calcination of ZLDH. This shift could be attributed to the absence of hydrogen bonding between the hydroxyl groups in the LDH framework and the oxygen atoms in the nitrate anions interlayer [50]. Further, the NO_3_^−1^ band disappeared, indicating the absence of lamellar structure. The Raman results are predictable with XRD data, and these characteristics are confirmed by the successful synthesis of the LDH nanocomposites.

#### 3.1.4. SEM and EDS

**Figure 4:**
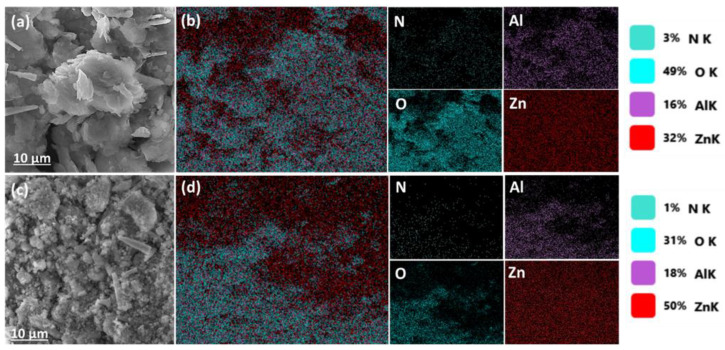
SEM images and corresponding EDS elemental maps of the powders: (**a**) SEM image of ZLDH, (**b**) EDS elemental map of ZLDH, (**c**) SEM image of CZLDH, and (**d**) EDS elemental map of CZLDH.

Figure 4 shows the morphological SEM images and the EDS mapping of the LDH nanocomposites. Figure 4a shows the aggregated smooth flakes-like shape (typical for LDHs) with poorly defined edges of ZLDH nanocomposites. As can be seen from the SEM image of the calcined LDH (CZLDH) in Figure 4c, the crystallinity was obviously increased after calcination, and this is perfectly compatible with XRD results.

For EDS images, Figure 4b,d, the elements (N, O, Al, Zn) are evenly distributed, and there is no discernible compositional distribution at this macroscale. Moreover, the uniformity of elements was clearly observed and suggested the successful preparation of LDH nanocomposites. The oxygen percent was reduced in the CZLDH nanocomposite, indicating that the water molecules in interlayer spacing are evaporated after calcination at high temperatures. In addition, the existence of metal oxides and spinel phases after annealing the ZnAl–LDH nanocomposite led to a high overall atomic percent of Al and Zn [37]. This observation is consistent with XRD results.

### 3.2. Characterization of ZLDH and CZLDH Coating

#### 3.2.1. SEM

**Figure 5:**
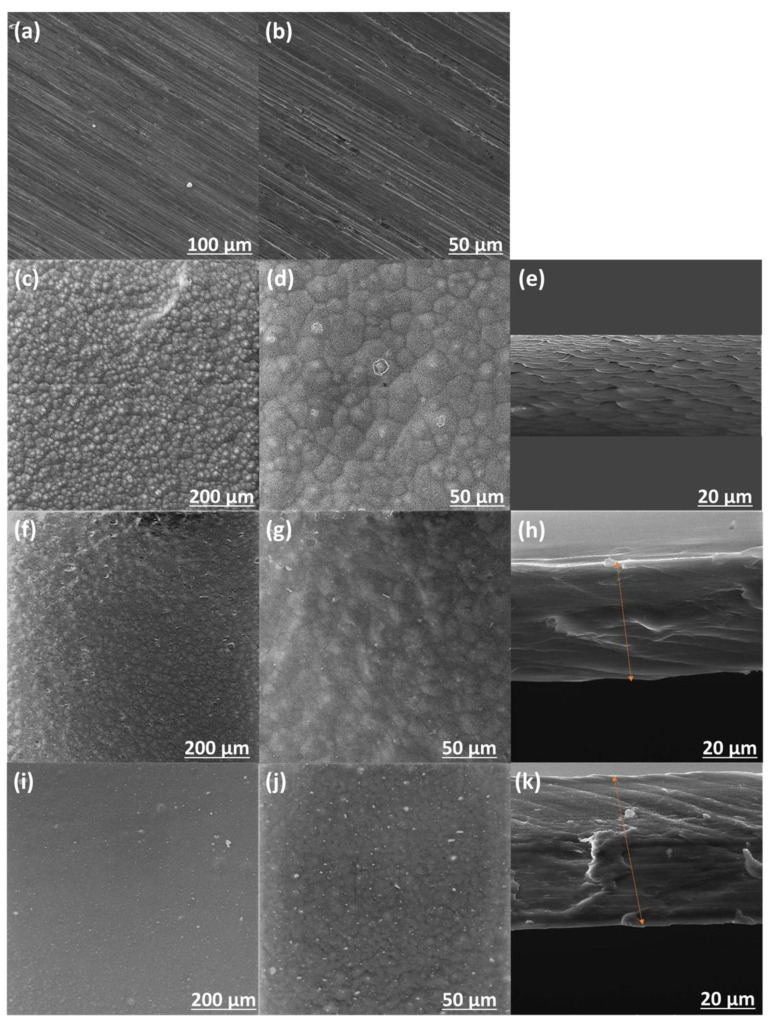
SEM images of the surface (**a**–**d**,**f**,**g**,**i**,**j**) and cross-section (**e**,**h**,**k**) of coatings on mild steel substrates: (**a**,**b**) bare substrate, (**c**–**e**) P-PVDF, (**f**–**h**) ZLDH/PVDF, and (**i**–**k**) CZLDH/PVDF.

SEM was employed to further analyze the surface morphology of the prepared coatings. Figure 5 shows the morphologies of the surface and cross-section of the P-PVDF, ZLDH/PVDF, and CZLDH/PVDF coatings. Figure 5a,b displays the surface of the uncoated polished mild steel substrate at different magnifications. The surface of the P/PVDF coating is shown in Figure 5c,d, and the lizard-skin-like morphology was distinguished. Further, the pores were distinctly observed in the cross-section of the P/PVDF coating in Figure 5e. Unfortunately, these pores could capture chloride ions and then sequentially reach the surface of the mild steel. After incorporating the less amount of LDH nanocomposites (2% wt.) into the polyvinyl difluoride matrix, it can be clearly seen that the lizard-skin effect of the surface was reduced (Figure 5f,g,i,j). Compared with the P-PVDF coating, the cross-section of the ZLDH/PVDF and CZLDH/PVDF coatings exhibits a compact and dense film, as shown in Figure 5h and Figure 5k, respectively, indicating the good interfacial interaction between the LDH nanocomposites (inorganic filler) and polyvinyl difluoride (organic matrix). The orange arrow lines in Figure 5h,k illustrate the integrity and uniformity of the coatings as observed in the cross-sectional images.

#### 3.2.2. Water Contact Angle

**Figure 6:**
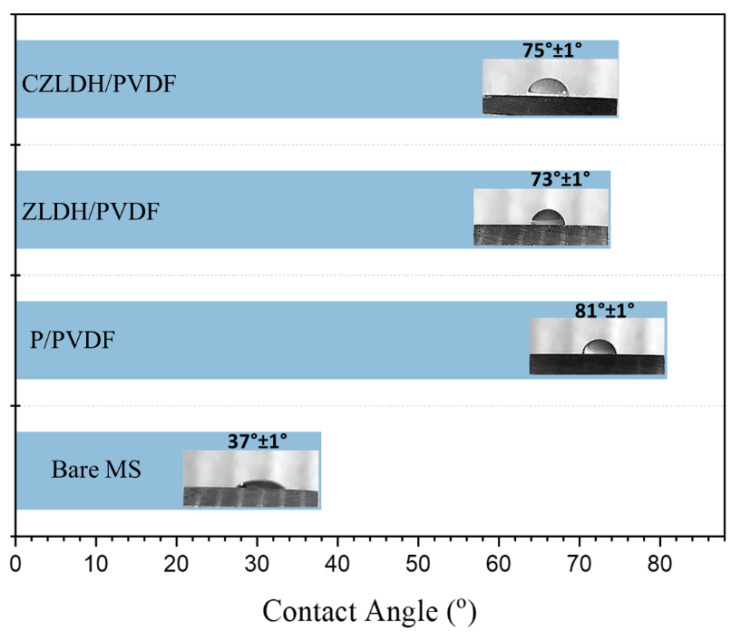
Contact angle measurements of bare and coated samples on a mild steel substrate.

The measurements of the water contact angle (WCA) were used to determine the properties of the coating surface. When the contact angle is below 90 degrees, the surface of the coatings is hydrophilic; otherwise, it is hydrophobic [51]. Figure 6 shows the water contact angle of the uncoated mild steel and the coated samples with the absence and presence of the LDH nanocomposites. It was observed that the WCA of the composite coatings decreased after the incorporation of the LDH nanocomposites compared to the P-PVDF coating. However, the WCA of the CZLDH/PVDF coating increased by around 2 degrees. The slight increase in WCA observed for the CZLDH/PVDF coating after calcination can be attributed to changes in both surface chemistry and surface roughness [52]. Upon calcination, the removal of interlayer water molecules and anions from the LDH structure leads to a transformation into a mixed metal oxide with altered surface properties. This change reduces the surface energy of the material due to the exposure of metal oxide sites, which are less hydrophilic compared to the hydroxyl-rich surface of non-calcined LDHs. Additionally, the calcination process may induce slight changes in the surface morphology of the CZLDH particles, potentially increasing surface roughness. These combined factors contribute to the slight increase in WCA observed for the CZLDH/PVDF coating. These findings are consistent with XRD and SEM results.

#### 3.2.3. Adhesion Test

**Figure 7:**
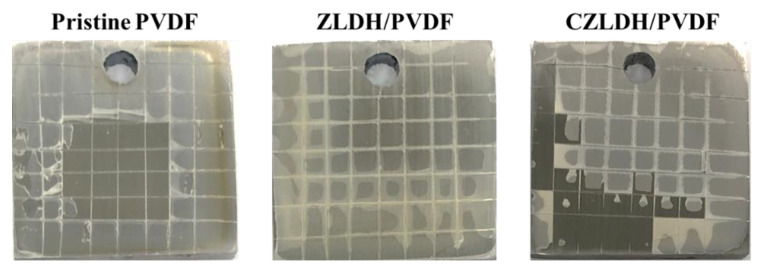
Optical images of the coated samples after a cross-cut tape test.

Adhesion is a critical factor for the durability and long-term performance of protective coatings. Poor adhesion can allow corrosive species or by-products of corrosion to penetrate beneath the coating layer, particularly at damaged sites. This can lead to undercutting, flaking, or complete detachment of the coating from the metal substrate [53]. Figure 7 shows the cross-cut tape test results for the coated samples: P/PVDF, ZLDH/PVDF, and CZLDH/PVDF. The ZLDH/PVDF coating exhibited no peeling or delamination on the cross-cut surface, demonstrating excellent adhesion to the mild steel substrate. This enhanced adhesion can be attributed to the incorporation of ZLDH nanocomposites, which improve the interface bonding. In contrast, significant delamination was observed for both the pristine PVDF (P-PVDF) and CZLDH/PVDF coatings, indicating poor adhesion and reduced coating stability.

### 3.3. Electrochemical Performance of the PVDF Polymer Composites

#### 3.3.1. Corrosion Behavior Analysis Through Polarization Curves

The potentiodynamic polarization data presented in Table 2 reveal important insights into the corrosion behavior of the studied coatings. The bare substrate, as depicted in Table 2, exhibits a notably negative corrosion potential E_corr_ of −828 mV vs. SCE, indicating a high susceptibility to corrosion. Furthermore, it demonstrates the highest corrosion current density i_corr_ among the coatings, quantified at 10.20 µA/cm^2^.

**Figure 8:**
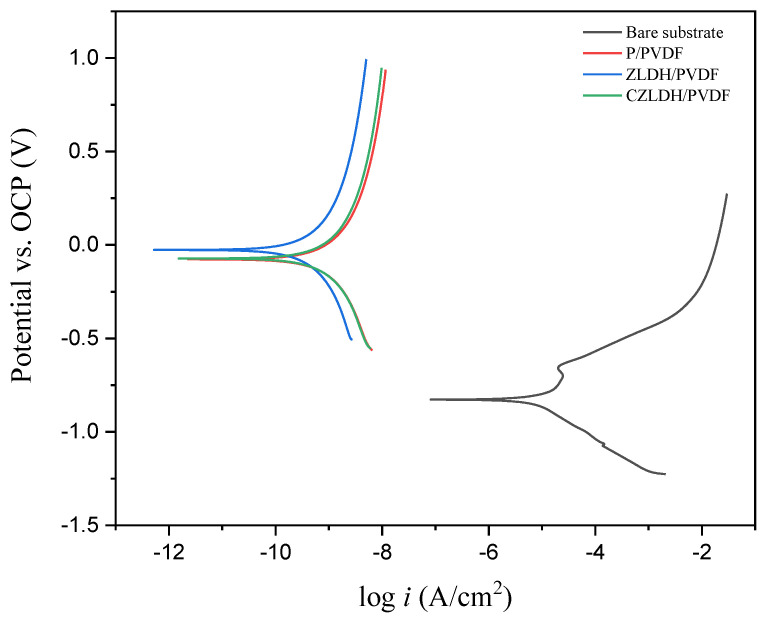
Potentiodynamic polarization PDP of the bare substrate and PVDF coating composites.

In contrast, the P/PVDF coating displays a less negative corrosion potential E_corr_ of −82.90 mV vs. SCE compared to the bare substrate, signifying some level of corrosion protection, as shown in Figure 8. However, its corrosion current density i_corr_ is notably higher, quantified at 16.40 × 10^−3^ µA/cm^2^, suggesting a moderate corrosion rate. These results indicate that while PVDF offers initial corrosion resistance, it is not highly effective in preventing long-term corrosion.

The potentiodynamic polarization data for the ZLDH/PVDF coating stand out with a significantly less negative corrosion potential E_corr_ of −29.60 mV vs. SCE compared to both the bare substrate and the P/PVDF coating. This signifies superior corrosion protection. Furthermore, it showcases the lowest corrosion current density i_corr_ among the coatings, quantified at 8.980 × 10^−4^ µA/cm^2^, indicative of the lowest corrosion rate.

In the case of the CZLDH/PVDF coating, as shown in Table 2, it exhibits a corrosion potential E_corr_ between that of P/PVDF and ZLDH/PVDF, implying intermediate corrosion protection. The corrosion current density i_corr_ of CZLDH/PVDF falls between that of P-PVDF and ZLDH/PVDF coatings, quantified at 13.40 × 10^−3^ µA/cm^2^. This suggests a moderate corrosion rate for CZLDH/PVDF, which is better than P-PVDF but not as effective as ZLDH/PVDF in preventing corrosion.

#### 3.3.2. Long-Term Corrosion Resistance of Coatings

**Figure 9:**
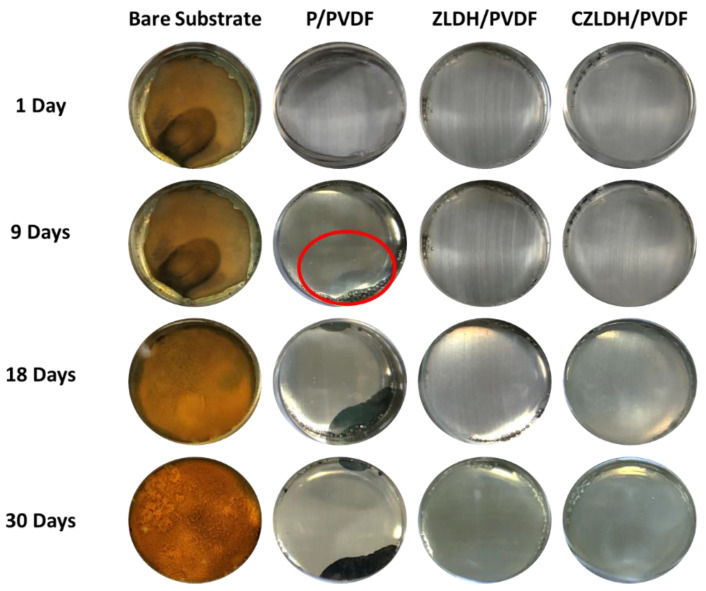
Optical photographs of the bare substrate, pristine PVDF, ZLDH/PVDF, and CZLDH/PVDF coatings in 3.5% NaCl solution for long-term immersion. The red circle highlights the blister caused by the initiation of corrosion on the P/PVDF coating after 9 days of immersion in NaCl.

**Figure 10:**
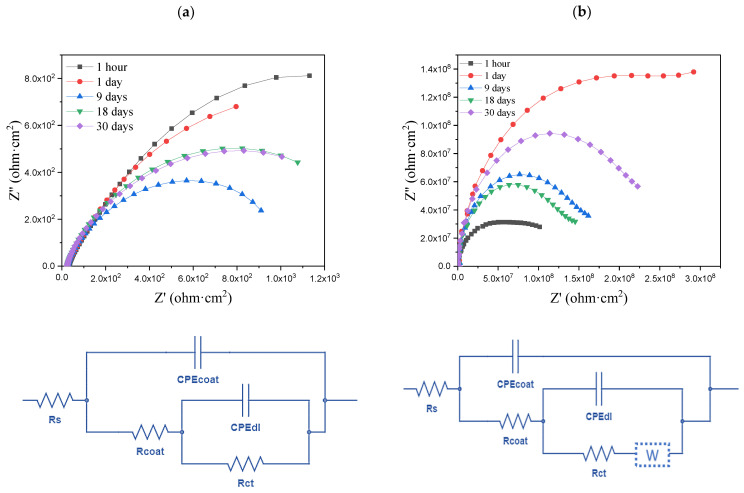

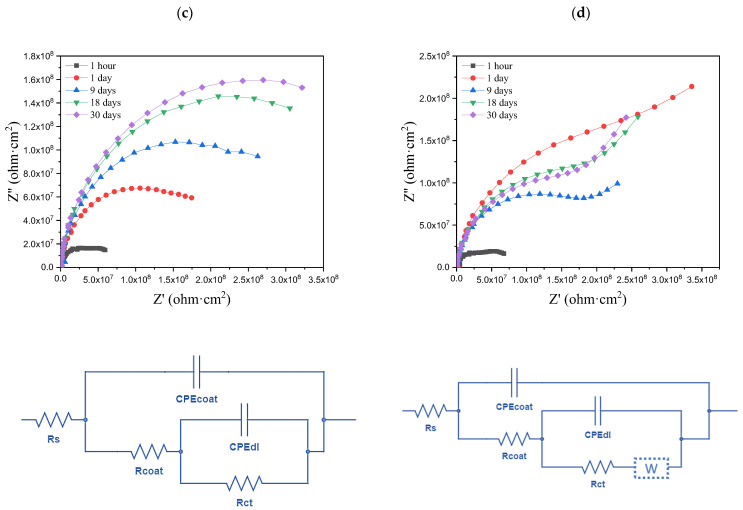
Nyquist plots with the respective equivalent circuits of (**a**) bare substrate, (**b**) P/PVDF, (**c**) ZLDH/PVDF, and (**d**) CZLDH/PVDF coating.

The long-term immersion results of the bare substrate, pristine PVDF, ZLDH/PVDF, and CZLDH/PVDF coatings in 3.5% NaCl solution are analyzed based on the optical photographs Figure 9, the electrochemical impedance spectroscopy data (Table 3), and the Nyquist Plots with the respective equivalent circuits (Figure 10). The presence or absence of key circuit elements, such as the Warburg impedance (W), highlights critical differences in the structure and diffusion behavior of the coatings.

The bare substrate displayed the most severe degradation among all samples. As shown in Figure 9, significant rust formation and corrosion occurred as early as one day of immersion, with the steel surface completely covered in corrosion products by 30 days. The EIS results further support this observation, as the impedance values remained consistently low throughout the test period. The equivalent circuit (Figure 10) for the bare substrate includes only the solution resistance (Rs) and double-layer capacitance (CPE_dl_) without the Warburg impedance element, indicating a direct interaction between the steel surface and the electrolyte. The polarization resistance (Rp) decreased steadily over time, dropping to 1.68 × 10^3^ Ω·cm^2^ after 30 days. This behavior confirms that the absence of a protective barrier led to continuous electrochemical activity and accelerated corrosion [54].

The pristine PVDF coating shows limited corrosion resistance due to its weak adhesion and susceptibility to water uptake. The optical images in Figure 9 reveal that the coating remained intact in the early stages; however, blistering and delamination began appearing after 9 days of immersion (indicated by the red circle in Figure 9), progressing further by 30 days. These visual observations align with the EIS results, where the equivalent circuit (Figure 10) includes a Warburg impedance element (W) alongside the coating constant phase element (CPE_coat_) and double-layer capacitance (CPE_dl_). The presence of the Warburg impedance reflects diffusion-controlled processes occurring through defects in the PVDF coating. Initially, the coating exhibited high resistance values, with R_1_ reaching 2.34 × 10⁸ Ω·cm^2^ at 1 day; however, the resistance decreased significantly to 1.86 × 10⁸ Ω·cm^2^ after 30 days. The increase in the coating capacitance values (CPE_coat_) indicates water adsorption and electrolyte penetration, which compromised the barrier properties over time [55]. These findings confirm that while pristine PVDF provides a temporary physical barrier, its poor adhesion and increased porosity limit its long-term corrosion protection.

The ZLDH/PVDF coating exhibited the best overall performance, maintaining its structural and electrochemical stability throughout the 30-day immersion period. As shown in Figure 9, the optical images reveal no signs of delamination, blistering, or rust formation on the coating surface, even after prolonged immersion. The EIS data further reinforce these observations, with the ZLDH/PVDF coating exhibiting the highest impedance values among all samples. The equivalent circuit (Figure 9) includes only CPE_coat_ and CPE_dl_, with no Warburg impedance component, indicating the absence of diffusion-controlled processes. The polarization resistance steadily increased to 8.52 × 10⁸ Ω·cm^2^ after 30 days, reflecting the excellent barrier properties of the coating. The superior performance of ZLDH/PVDF is attributed to the intact lamellar structure of ZLDH nanofillers, which enhance adhesion, reduce coating porosity, and create a twisted path that effectively impedes chloride ion diffusion. This combination of structural and barrier properties ensures long-term corrosion resistance and mechanical stability.

The CZLDH/PVDF coating, in contrast, demonstrated intermediate performance, exhibiting some signs of degradation over time. The optical images (Figure 9) show minor delamination and localized rust formation by 9 days, which became more pronounced at 30 days. The EIS results provide further insight into the coating’s behavior, with the equivalent circuit (Figure 10) including a Warburg impedance element alongside the coating and double-layer capacitance components. The presence of W suggests partial diffusion of chloride ions through micro-defects in the coating. Although the coating resistance started at a relatively high value, it plateaued at 1.91 × 10⁸ Ω·cm^2^ after 30 days, indicating a reduction in long-term protective performance compared to ZLDH/PVDF. The higher CPE_coat_ values for CZLDH/PVDF reflect increased water uptake and porosity due to the rigid and less compatible nature of CZLDH particles. This reduced performance is attributed to the change of structure during calcination, which compromises interfacial adhesion and barrier efficiency (Figure S1).

#### 3.3.3. Scratch Test

**Figure 11:**
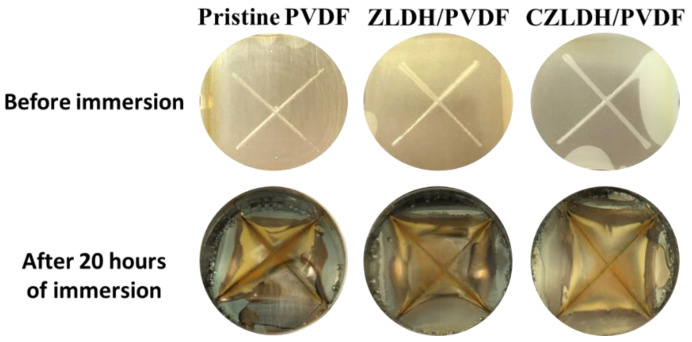
Scratch test of the coated samples before and after immersing in 3.5% NaCl.

The scratch test results provide insights into the adhesion performance of the coatings on the mild steel substrate. The top row of Figure 11 shows the coated samples before exposure to NaCl, while the bottom row shows the same samples after the scratch test in a corrosive 3.5% NaCl solution.

The pristine PVDF coating shows significant peeling and delamination at the cross-cut regions after the scratch test. This poor adhesion performance is due to the inherent lack of chemical compatibility between PVDF and the mild steel substrate, leading to weak interfacial bonding. This allows aggressive chloride ions to penetrate and further undercut the coating. The ZLDH/PVDF coating exhibits no visible peeling or delamination after the scratch test, indicating excellent adhesion. The superior performance can be attributed to the intact lamellar structure of ZLDH. The CZLDH/PVDF coating shows moderate adhesion performance, with minor peeling observed around the scratch lines. This intermediate behavior is due to the structural transformation of ZLDH into a rigid oxide (CZLDH) during calcination. The calcined form loses its lamellar flexibility, leading to weaker interfacial bonding and slightly increased porosity compared to ZLDH/PVDF [26].

**Figure 12:**
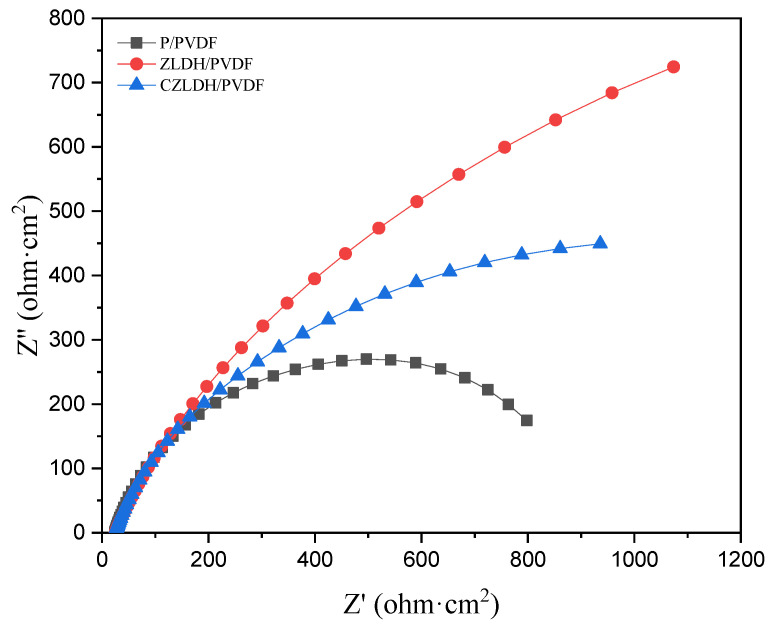
Nyquist plot of scratch test of the coated samples.

The EIS was performed for all the samples after the scratch test to provide valuable information about the corrosion resistance of the coatings. The Nyquist plot in Figure 12 shows the impedance response of the bare substrate, P/PVDF, ZLDH/PVDF, and CZLDH/PVDF coatings after 15 days of immersion in 3.5% NaCl. The P/PVDF coating shows a slightly larger semicircle compared to the bare substrate, reflecting improved barrier properties. However, the relatively low impedance response suggests that the coating is insufficient in preventing chloride ion penetration over time due to its porosity and poor adhesion. The ZLDH/PVDF coating displays the largest semicircle, corresponding to the highest impedance among all tested samples. On the other hand, the CZLDH/PVDF coating exhibits an intermediate impedance response, with a semicircle smaller than ZLDH/PVDF but larger than P/PVDF. While the calcined form CZLDH contributes to some level of barrier protection, the loss of the lamellar structure during calcination reduces its ability to impede ion diffusion effectively.

### 3.4. Optimum Coating

In order to determine the optimum coating, the amount of LDH composite, curing time, and curing temperature corresponding to the lowest corrosion current density were studied. The amount of inorganic filler (ZLDHs), curing time, and curing temperature were selected as (2%, 4%, 6% wt.), (1, 2, 3 h), and (135 °C, 155 °C, 175 °C), respectively.

The results from both experimental sections and the applied tests, including adhesion, water contact angle (WCA), open circuit potential (OCP), electrochemical impedance spectroscopy (EIS), and Tafel polarization, demonstrate that the ZLDH composite exhibits superior properties compared to P-PVDF and CZLDH composite. These properties include enhanced self-healing ability, robust adhesion, and exceptional corrosion-resistant coating performance. Accordingly, the ZLDH/PVDF coating achieves the lowest corrosion current density (indicative of optimal corrosion protection) when prepared under optimized parameters, as shown in Table 1, which details the variables and their levels.

**Figure 13:**
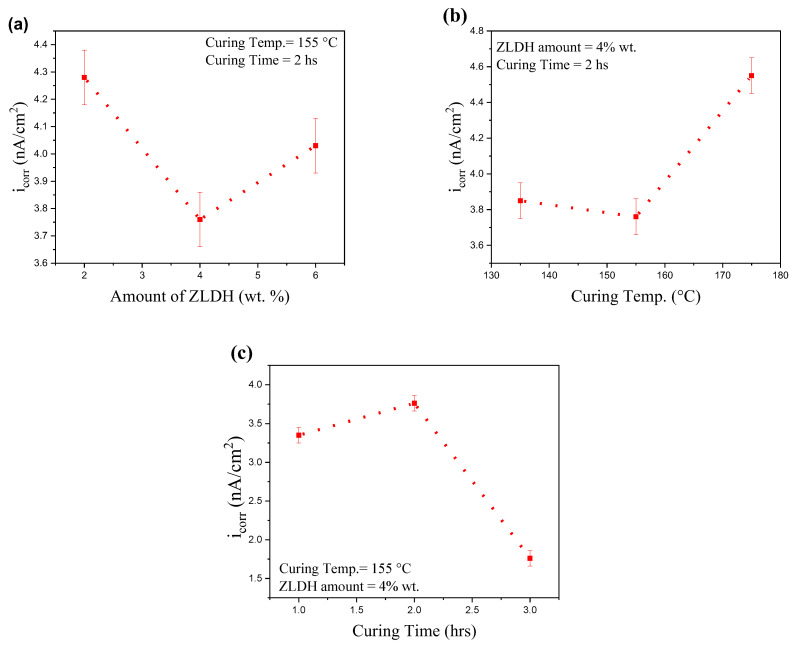
Plots showing the effect of the variable study on coating performance: (**a**) effect of ZLDH amount, (**b**) effect of curing temperature, and (**c**) effect of curing time.

All prior experiments were conducted at 155 °C for 2 h with an LDH content of 2%. Figure 13 illustrates the influence of ZLDH content, curing temperature, and curing time on corrosion current density, assessed via the Tafel test on the coated samples. Figure 13a shows the relationship between corrosion current density and ZLDH concentration, revealing that an optimal ZLDH content of 4% achieves the lowest corrosion current density (i_corr_). This ideal ZLDH amount likely aligns well with the pore size and volume of the PVDF coating matrix.

Curing temperature, varied between 135 °C and 175 °C, also impacts i_corr_, as shown in Figure 13b. At 155 °C, the i_corr_ value is minimized compared to other temperatures. This optimal temperature is closely associated with the DMF solvent’s boiling point of 153 °C, which allows for complete solvent evaporation from the coating. Higher curing temperatures risk creating defects due to the PVDF melting range of 170–185 °C, as previously reported [56]. Additionally, Figure 13c shows that extended curing time at 155 °C further reduces i_corr_, likely due to enhanced interactions between the polymer and ZLDH, which strengthen the cross-linked structure of the coating. In conclusion, based on the results presented in Figure 13, it seems that 4% wt., 3 h, and 155 °C exhibit the lowest corrosion current density.

### 3.5. Mechanism

The superior performance of ZLDH/PVDF compared to P/PVDF and CZLDH/PVDF coatings can be explained by examining the interplay of structure, morphology, and interfacial properties that govern the coatings’ anti-corrosion behavior. Despite both ZLDH and CZLDH containing zinc elements, their contrasting performance lies in their structural integrity and the resulting influence on the coating barrier efficiency.

The key factor contributing to ZLDH/PVDF’s performance is the intact lamellar structure of ZLDH, which directly enhances the coating’s compactness, adhesion, and barrier properties. The lamellar architecture of ZLDH acts as a highly efficient filler, creating a tortuous diffusion path for chloride ions and significantly impeding their penetration through the coating to the metal substrate [57]. This structural feature reduces the permeability of the coating, preventing direct contact between the aggressive electrolyte and the underlying steel surface. Previous studies have highlighted that LDHs with an intact lamellar structure improve mechanical reinforcement and effectively block ion diffusion in polymer matrices, thus enhancing corrosion resistance [58,59].

In contrast, CZLDH/PVDF exhibited a moderate performance, primarily due to the collapse of the lamellar structure during calcination. While calcination converts ZLDH into a crystalline oxide form with spinel phases, it also eliminates the interlayer water and hydroxyl groups, which play a critical role in enhancing interfacial bonding with the PVDF matrix. The resulting CZLDH particles are rigid and less compatible with the polymer, leading to weaker adhesion and the formation of micro-defects within the coating. These defects compromise the barrier effect and allow for partial chloride ion diffusion [60]. The observed Warburg impedance in CZLDH/PVDF further supports this, indicating diffusion-controlled processes that arise due to structural defects. Similar findings have been reported in systems where calcined LDHs lost their efficiency as barrier fillers due to reduced structural flexibility and surface energy changes [21,61].

The interfacial interaction between the LDH nanofillers and the PVDF matrix is another critical factor influencing coating performance [62]. ZLDH, owing to its hydroxyl-rich and hydrophilic surface, establishes stronger interfacial bonding with the PVDF matrix. This enhanced cohesion results in a more uniform dispersion of the filler and eliminates voids or pathways that could facilitate ion transport [63]. The reduction in the lizard-skin effect, as observed in the morphology of ZLDH/PVDF, further confirms the improved filler–polymer interaction. In contrast, CZLDH’s calcined surface has a lower surface energy due to the loss of functional groups, reducing its ability to bond effectively with the PVDF matrix. The resulting poorer dispersion and weaker interfacial bonding explain the presence of micro-defects and the slightly higher porosity observed in CZLDH/PVDF coatings.

Another influencing factor is the surface wettability of the coatings. Although ZLDH slightly reduces the water contact angle, the improved hydrophilicity benefits the formation of a compact, defect-free coating structure. This compactness outweighs any reduction in hydrophobicity by preventing water and chloride ion ingress. On the other hand, CZLDH/PVDF shows a slight increase in hydrophobicity due to calcination-induced changes in surface energy. However, this increase is insufficient to overcome the structural defects and weaker interfacial bonding, which ultimately limit the coating’s performance. It is worth noting that hydrophobicity alone cannot guarantee superior corrosion resistance, particularly when the coating lacks structural integrity, as reported in previous studies on polymer-based anti-corrosion coatings [26].

In summary, the superior performance of ZLDH/PVDF coatings arises from the synergistic effects of the intact lamellar structure, which enhances barrier efficiency, the strong interfacial bonding with the PVDF matrix, and the reduced structural porosity that eliminates pathways for ion diffusion. These properties combine to form a compact and highly effective protective layer against corrosion. In contrast, CZLDH/PVDF’s reduced performance is attributed to the structural collapse during calcination, leading to weaker interfacial bonding, increased porosity, and partial diffusion effects. P/PVDF, lacking both structural reinforcement and strong interfacial cohesion, provides only limited protection. These findings align with prior research where LDH-based coatings demonstrated enhanced corrosion resistance due to their ability to reduce ion permeability and strengthen coating integrity [64].

## 4. Conclusions

This study comprehensively evaluated the corrosion resistance, mechanical performance, and structural properties of poly(vinylidene fluoride) (PVDF)-based coatings incorporating zinc–aluminum layered double hydroxide (ZLDH) and its calcined form (CZLDH). The investigation involved structural characterizations (FTIR, XRD, Raman, SEM/EDS), surface wettability (WCA), adhesion tests, scratch tests, potentiodynamic polarization, and electrochemical impedance spectroscopy (EIS) to identify key factors influencing coating performance.

- SEM and EDS analysis revealed significant morphological differences. The ZLDH/PVDF coating exhibited a dense and compact structure, with well-dispersed nanofillers and minimal defects, which improved interfacial adhesion. In contrast, CZLDH/PVDF displayed micro-defects due to the rigid and less flexible calcined fillers, while P-PVDF showed visible pores and a lizard-skin texture that facilitated chloride ion diffusion;
- WCA measurements indicated that the incorporation of ZLDH slightly reduced the hydrophobicity of the PVDF coating due to the presence of hydroxyl-rich ZLDH fillers. However, the slight increase in WCA for CZLDH/PVDF resulted from reduced surface energy after calcination;
- Adhesion tests show that ZLDH/PVDF exhibited superior adhesion with no peeling or delamination on the cross-cut surface, highlighting strong interfacial bonding. Conversely, CZLDH/PVDF displayed moderate adhesion, while P/PVDF suffered significant delamination due to weak bonding with the mild steel substrate;
- Scratch tests further validated the adhesion results, with ZLDH/PVDF showing no visible damage or detachment after scratching and validated by EIS after 15 days with the highest corrosion-resistant sample. CZLDH/PVDF displayed minor peeling, while P/PVDF exhibited significant failure, underscoring the critical role of the intact ZLDH lamellar structure in enhancing mechanical stability;
- Potentiodynamic polarization analysis demonstrated that ZLDH/PVDF achieved the lowest corrosion current density (8.98 × 10⁻⁴ µA/cm^2^) and the most positive corrosion potential (−29.60 mV), confirming its superior corrosion resistance. CZLDH/PVDF displayed intermediate corrosion protection, while P-PVDF showed the highest corrosion rate due to its porous and defective structure;
- Long-term immersion and EIS studies revealed the sustained performance of ZLDH/PVDF over 30 days in NaCl solution. ZLDH/PVDF exhibited the highest impedance values and the absence of Warburg impedance, reflecting its exceptional barrier properties. In contrast, CZLDH/PVDF shows moderate impedance with evidence of diffusion processes;
- Optimization of coating parameters demonstrated that the ZLDH/PVDF coating achieved the best corrosion resistance under the following conditions: 4 wt.% ZLDH content, 155 °C curing temperature, and 3 h curing time. These optimized parameters resulted in the lowest corrosion current density and enhanced coating integrity.

In conclusion, the synergistic effects of the intact lamellar structure, strong interfacial bonding, and compact morphology of ZLDH/PVDF resulted in superior corrosion resistance, mechanical robustness, and long-term stability. The findings highlight the importance of maintaining LDH structural integrity for the development of advanced anti-corrosion polymer-based coatings for metallic substrates in aggressive environments.

## Figures and Tables

**Table 1:** The levels of the selected factors to optimize the ZLDH coatings.

Factors	1	2	3
ZLDH amount (wt.%)	2%	4%	6%
Curing temperature (°C)	135	155	175
Curing time (hour)	1	2	3

**Table 2:** Potentiodynamic polarization data of the coated samples in 3.5%NaCl solutions at RT.

Specimens	E_corr_ (mV vs. SCE)	i_corr_ (µA/cm^2^)
Bare substrate	−828	10.20
P/PVDF	−82.90	16.40 × $10^{-3}$
ZLDH/PVDF	−29.60	8.980 × $10^{-4}$
CZLDH/PVDF	−72.60	13.40 × $10^{-3}$

**Table 3:** The electrochemical impedance spectroscopy data EIS of the bare steel and polymeric coated samples for 30 days of immersing in 3.5% NaCl.

Sample	Immersion Time	R_s_, Ω·cm^2^	CPE1		R_1_, Ω·cm^2^	CPE2		R_2_, Ω.cm^2^	ZW, S × s^1/2^	Rp, Ω·cm^2^
			Y_1_	a_1_		Y_2_	a_2_			
CS	1 h	31.78	4.26 × 10^−4^	8.01 × 10^−1^	4.14 × 10^2^	3.74 × 10^−4^	7.28 × 10^−1^	2.13 × 10^3^		2.54 × 10^3^
	1 d	21.90	6.38 × 10^−4^	7.34 × 10^−1^	3.06 × 10	3.98 × 10^−4^	6.95 × 10^−1^	2.37 × 10^3^		2.40 × 10^3^
	9 d	26.16	2.41 × 10^−4^	8.58 × 10^−1^	2.73 × 10^2^	4.13 × 10^−4^	6.75 × 10^−1^	8.17 × 10^2^		1.09 × 10^3^
	18 d	24.48	2.30 × 10^−4^	8.96 × 10^−1^	1.84 × 10^2^	4.24 × 10^−4^	5.99 × 10^−1^	1.43 × 10^3^		1.61 × 10^3^
	30 d	24.12	2.68 × 10^−4^	8.94 × 10^−1^	1.90 × 10^2^	5.25 × 10^−4^	5.69 × 10^−1^	1.49 × 10^3^		1.68 × 10^3^
P/PVDF	1 h	19.00	7.38 × 10^−10^	9.55 × 10^−1^	2.50 × 10^7^	7.79 × 10^−9^	3.70 × 10^−1^	1.60 × 10^8^		1.85 × 10^8^
	1 d	29.00	9.50 × 10^−10^	9.56 × 10^−1^	2.34 × 10^8^	1.17 × 10^−8^	9.61 × 10^−1^	4.08 × 10^4^	9.82 × 10^−9^	2.34 × 10^8^
	9 d	12.00	7.34 × 10^−10^	9.75 × 10^−1^	1.01 × 10^7^	3.18 × 10^−9^	8.15 × 10^−1^	3.97 × 10^7^	3.77 × 10^−8^	4.98 × 10^7^
	18 d	25.00	8.03 × 10^−10^	9.68 × 10^−1^	1.15 × 10^8^	2.11 × 10^−8^	1.00	1.44 × 10^7^	4.65 × 10^−8^	1.29 × 10^8^
	30 d	27.00	8.25 × 10^−10^	9.65 × 10^−1^	1.86 × 10^8^	5.71 × 10^−11^	9.40 × 10^−1^	1.09 × 10^5^	2.09 × 10^−8^	1.86 × 10^8^
ZHDL/PVDF	1 h	19.00	1.22 × 10^−9^	9.30 × 10^−1^	1.71 × 10^4^	1.10 × 10^−8^	2.34 × 10^−1^	1.40 × 10^8^		1.40 × 10^8^
	1 d	28.00	9.13 × 10^−10^	9.58 × 10^−1^	7.37 × 10^3^	3.57 × 10^−6^	2.36 × 10^−1^	4.53 × 10^8^		4.53 × 10^8^
	9 d	21.00	7.01 × 10^−10^	9.80 × 10^−1^	1.04 × 10^4^	1.95 × 10^−9^	3.51 × 10^−1^	4.86 × 10^8^		4.86 × 10^8^
	18 d	36.00	8.83 × 10^−10^	9.62 × 10^−1^	2.57 × 10^5^	2.96 × 10^−9^	175.3 × 10^−3^	7.67 × 10^8^		7.67 × 10^8^
	30 d	27.00	8.55 × 10^−10^	9.63 × 10^−1^	1.93 × 10^4^	1.76 × 10^−9^	2.95 × 10^−1^	8.52 × 10^8^		8.52 × 10^8^
CZHDL/PVDF	1 h	21.00	7.73 × 10^−10^	9.56 × 10^−1^	7.96 × 10^3^	1.06 × 10^−8^	2.54 × 10^−1^	1.80 × 10^8^		1.80 × 10^8^
	1 d	15.00	7.20 × 10^−10^	9.58 × 10^−1^	1.73 × 10^8^	2.59 × 10^−9^	9.45 × 10^−1^	5.38 × 10^7^	5.34 × 10^−9^	2.27 × 10^8^
	9 d	19.00	6.44 × 10^−10^	9.68 × 10^−1^	1.47 × 10^8^	6.30 × 10^−10^	9.99 × 10^−1^	1.70 × 10^6^	1.02 × 10^−8^	1.49 × 10^8^
	18 d	14.00	7.74 × 10^−10^	9.53 × 10^−1^	1.67 × 10^8^	5.48 × 10^−10^	9.94 × 10^−1^	4.38 × 10^6^	6.47 × 10^−9^	1.72 × 10^8^
	30 d	18.00	7.84 × 10^−10^	9.53 × 10^−1^	1.91 × 10^8^	2.65 × 10^−9^	9.98 × 10^−1^	3.79 × 10^5^	5.37 × 10^−9^	1.91 × 10^8^

## Data Availability

The original contributions presented in this study are included in the article/Supplementary Material. Further inquiries can be directed to the corresponding author.

## Supplementary Materials

The following supporting information can be downloaded at: https://www.mdpi.com/article/10.3390/polym17030331/s1, Figure S1. BODE plots of (a,b) bare substrate, (c,d) P/PVDF, (e,f) ZLDH/PVDF, and (j,h) CZLDH/PVDF; Table S1. Crystallite size of the LDH composites and other parameters from the X-ray diffraction.
