# Supplementary material for: Synergistic Effects of Zn-Rich Layered Double Hydroxides on the Corrosion Resistance of PVDF-Based Coatings in Marine Environments
Source: Polymers (Basel). 2025 Jan 25;17(3):331. doi: 10.3390/polym17030331 (PMC11821123; doi:10.3390/polym17030331)
Supplement: Supplementary file 1 [file polymers-17-00331-s001.zip › polymers-3410367-supplementary.pdf]

Supplementary Materials

Synergistic Effects of Zn-Rich Layered Double Hydroxides on the Corrosion Resistance of PVDF-Based Coatings in Marine Environments

Hissah A. Alqahtani [a]*, Jwaher M. AlGhamdi [a*], Nuhu Dalhat Mu'azu [b]

[a] Department of Chemistry, College of Science, Imam Abdulrahman Bin Faisal University, 31451 Dammam, Saudi Arabia

[b] Department of Environmental Engineering, College of Engineering, Imam Abdulrahman Bin Faisal University, P.O. Box 1982, 31451 Dammam, Saudi Arabia
*Corresponding authors: hlqahtani@iau.edu.sa - jmalghamdi@iau.edu.sa

Table S1. Crystallite size of the LDH composites and other parameters from the X-ray diffraction.

CZLDH			ZLDH		
2Theta (deg)	FWHM (deg)	Crystallite Size D (nm)	2Theta (deg)	FWHM (deg)	Crystallite Size D (nm)
31.06	0.85	9.77	10.14	0.86	9.36
31.57	0.35	23.65	19.92	0.63	13.00
34.22	0.37	22.88	21.22	1.00	8.12
36.07	0.37	22.66	31.70	0.44	19.16
36.74	0.53	15.98	34.32	0.46	18.43
37.10	0.36	23.28	36.19	0.47	17.99
47.35	0.36	24.55	56.53	0.45	20.05
55.39	0.80	11.34			
56.42	0.34	26.80			
58.61	0.26	35.04			
58.99	0.89	10.28			
62.68	0.39	24.44			
64.97	0.88	10.73			
66.20	0.31	31.10			
66.20	0.35	27.59			
68.93	0.35	28.27			
72.43	0.33	30.15			
76.85	0.42	24.71			
89.4748	0.48	23.30			

Figure S1. BODE plots of (a,b) bare substrate, (c,d) P/PVDF, (e,f) ZLDH/PVDF, and (j.h) CZLDH/PVDF.

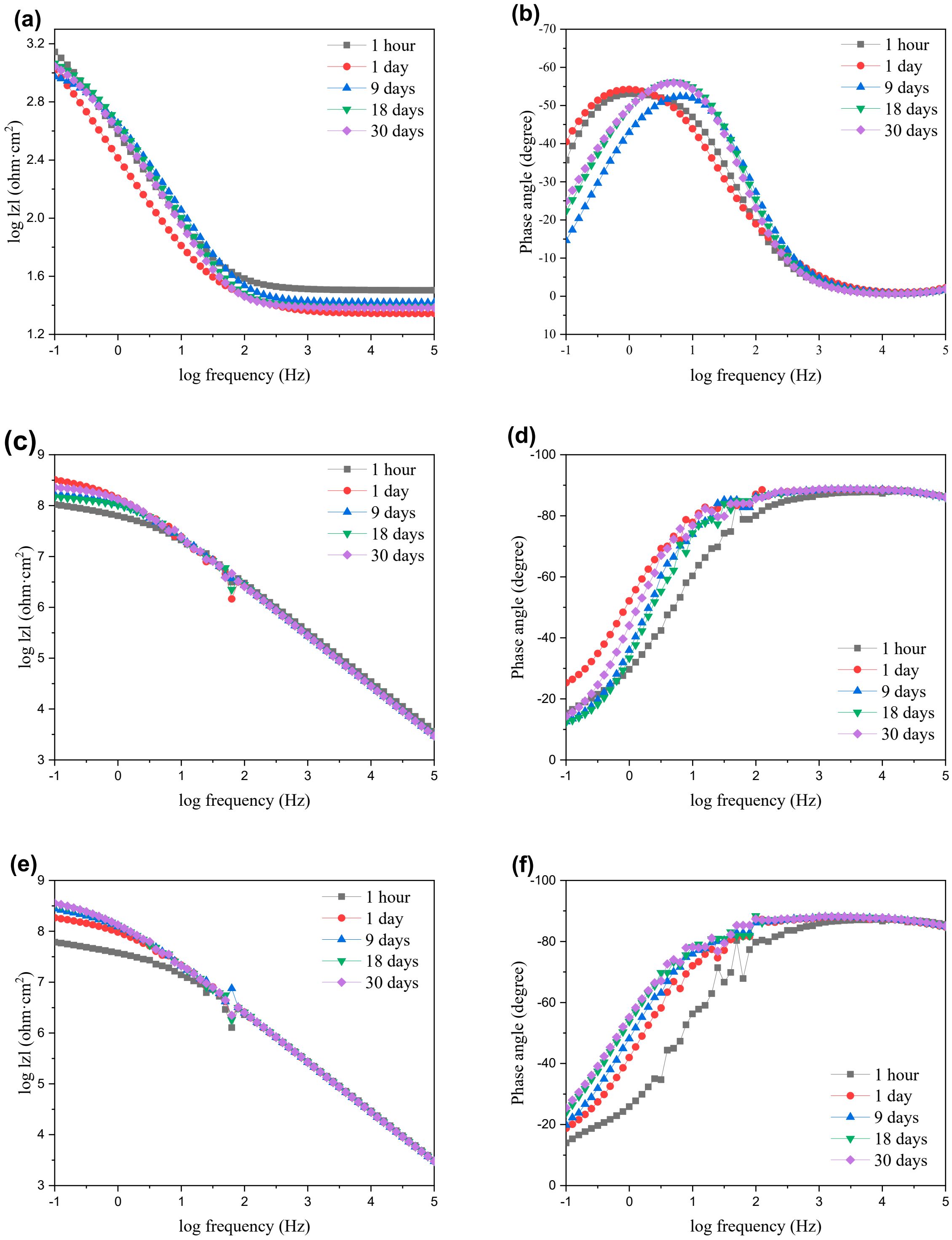

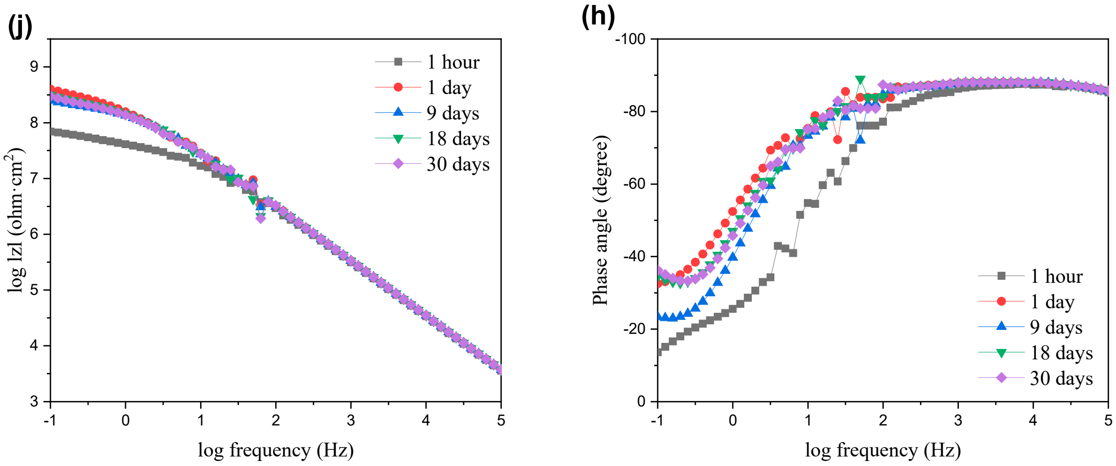
(j)
log lzl (ohm·cm2)
log frequency (Hz)
1 hour
1 day
9 days
18 days
30 days
9
8
7
6
5
4
3
-1
0
1
2
3
4
5
(h)
Phase angle (degree)
log frequency (Hz)
1 hour
1 day
9 days
18 days
30 days
-100
-80
-60
-40
-20
0
-1
0
1
2
3
4
5
